# Dysregulated iron metabolism and kidney stone risk: an epidemiological and experimental study

**DOI:** 10.1080/0886022X.2026.2631316

**Published:** 2026-03-03

**Authors:** Wenlong Wan, Dongfeng Yuan, Yang Xun, Xiao Yu

**Affiliations:** Department of Urology, Tongji Hospital of Tongji Medical College of Huazhong University of Science and Technology, Wuhan, Hubei, China

**Keywords:** Kidney stones, iron status, transferrin receptor, iron intake, NHANES

## Abstract

Kidney stone disease is a major global health burden with high recurrence, closely linked to oxidative stress. While iron, a redox-active metal, can exacerbate oxidative stress *via* the Fenton reaction, population evidence on its association with kidney stone risk is scarce. Based on data from 4,370 adults in the National Health and Nutrition Examination Survey (NHANES 2017–2018), we conducted survey-weighted multivariable logistic regression to assess the associations between iron status indicators and kidney stone risk, and used restricted cubic splines to examine potential dose-response relationships. Animal tissues were analyzed *via* IHC, Western blot, and biochemical assays. After adjustment, each 10% increase in transferrin saturation correlated with a 26% lower likelihood of kidney stones (odds ratio [OR] 0.74, 95% confidence interval [CI] 0.56–0.97, *p* = .04), while each 10 mg/day increase in dietary iron intake correlated with a 12% higher risk (OR 1.12, 95% CI 1.01–1.24, *p* = .036). Dose-response analysis revealed a U-shaped relationship for iron intake (inflection point: 11.7 mg/day). Animal experiments confirmed renal iron accumulation, elevated sTfR, and activated ferroptosis. This study first demonstrates a significant association between dysregulated iron metabolism and kidney stone risk, suggesting that systemic iron dysregulation and local renal ferroptosis may contribute to stone pathogenesis, offering new preventive insights.

## Introduction

Kidney stones represent one of the most prevalent conditions in urology. Epidemiological studies indicate a rising global prevalence of nephrolithiasis, with a five-year recurrence rate as high as 50%, establishing it as a significant public health burden worldwide [1–3].

Iron is an essential trace element yet also a potential toxic substance [4–6]. As a redox-active metal, excess iron can induce redox reactions, leading to increased levels of reactive oxygen species (ROS) and subsequent cellular inflammation and oxidative stress [7,8].

Iron metabolism dysregulation has been implicated in various diseases, such as liver fibrosis [9], Parkinson’s disease [10], diabetes mellitus [11], and dyslipidemia [12]. Substantial evidence suggests that the initiation and progression of kidney stones result from a series of pathological processes involving renal tubular epithelial cell inflammation, lipid metabolism disorder, and oxidative stress [13–15]. A recent case report suggested that iron injection might potentially induce kidney stone formation [16]. As a redox-active metal, excess iron-triggered oxidative stress and inflammation can damage renal tubular epithelial cells, creating a foundation for crystal adherence and stone formation. This mechanism aligns closely with the established pathological processes of cellular damage and inflammation in nephrolithiasis. Therefore, iron homeostasis imbalance may represent a novel pathological pathway in stone formation.

However, the relationship between iron load and the risk of kidney stone occurrence has been scarcely or never investigated. This study aims to explore and analyze the potential association between iron load and the risk of nephrolithiasis using multiple approaches, intending to provide new insights into the mechanisms of stone formation and clinical prevention strategies.

## Methods

### Study population

**Figure 1.**
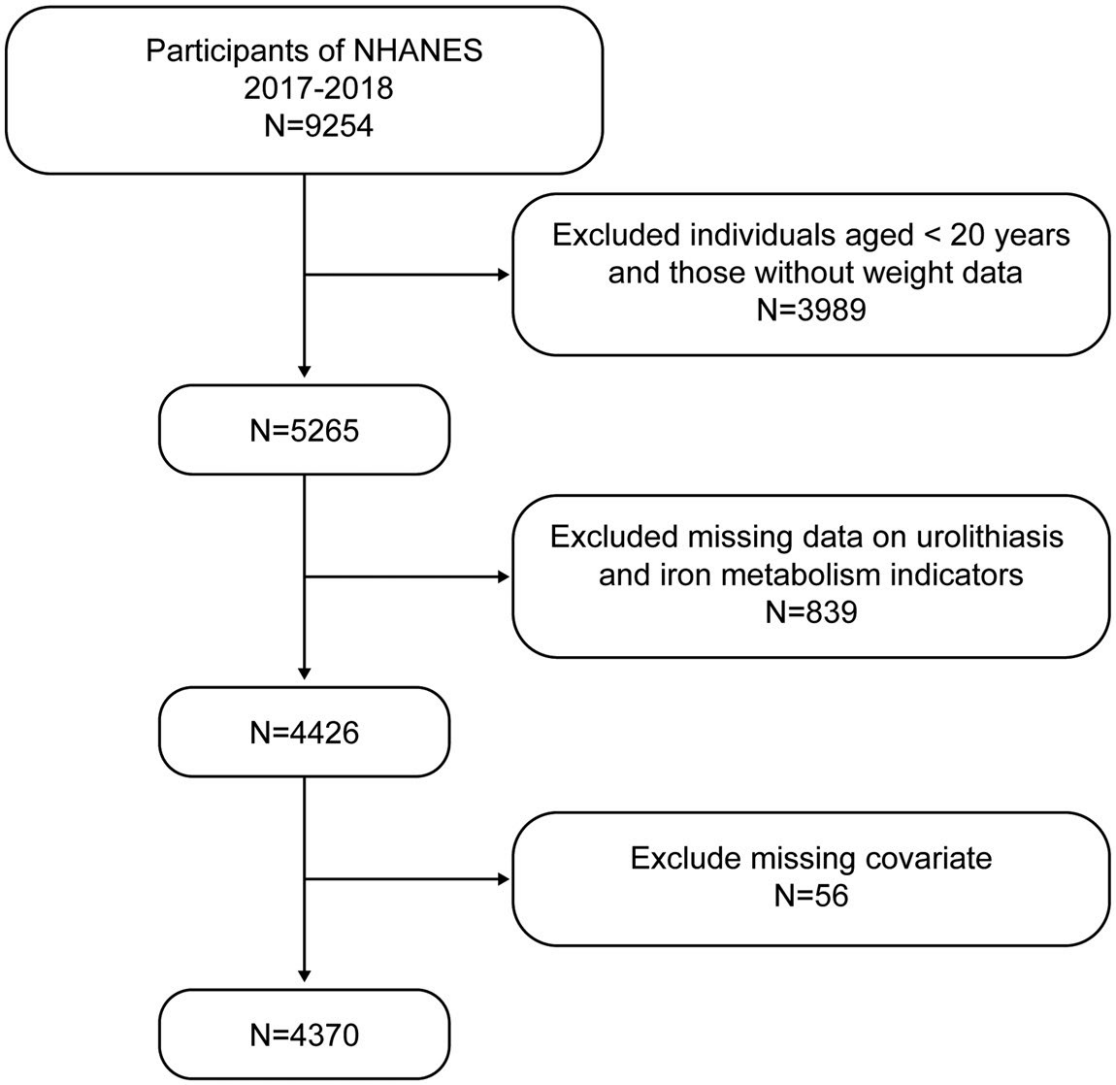
Study flowchart National Health and Nutrition Examination Survey, 2017–2018.

The NHANES is a multistage, complex study conducted by the National Center for Health Statistics to collect data on demographics, diet, socioeconomic status, and health-related issues, including screening data. For this study, data from the NHANES 2017–2018 cycle were utilized. Initially, 9,254 participants were available. After selecting 5,565 adults with complete sample weight data and subsequently excluding 839 participants with unknown kidney stone status or missing iron status data, along with 56 participants lacking data on important covariates such as BMI and smoking status, a final cohort of 4,370 participants was included in the analysis. These participants represent the non-institutionalized U.S. population aged 20 years and older. The included participants were largely comparable to the overall eligible population in terms of key demographic characteristics and primary exposure factors (Supplementary Table 1). The process of data consolidation and participant screening is illustrated in Figure 1. All participants provided written informed consent, and the data acquisition and analysis adhered to the NHANES study protocols.

### Study variables and results

The exposure of interest in this study was body iron load, assessed through multiple indicators: serum ferritin (SF), serum iron (SI), transferrin saturation (TSAT), soluble transferrin receptor (sTfR), hemoglobin, mean corpuscular hemoglobin, and mean daily dietary iron intake. Iron load indicators were measured using an improved high-performance liquid chromatography method with a photodiode array detector. Daily iron intake was calculated based on detailed dietary information collected from two 24-h dietary recalls. Total daily iron intake was defined as the sum of iron from food and iron from dietary supplements.

All statistical analyses strictly followed the NHANES analytical guidelines. To ensure national representativeness for the U.S. adult population, appropriate dietary day one sample weights (WTDRD1) and adjustments for the complex survey design variables (SDMVSTRA, SDMVPSU) were applied. Key covariates included sex, age, body mass index (BMI, kg/m^2^), race/ethnicity, smoking status, sedentary behavior, daily calcium intake, and the systemic inflammation response index (SIRI). Study participants were classified into either the kidney stone group or the non-kidney stone group based on their response to the interview question: ‘Have you ever had a kidney stone?’

### Animal experiments and animal ethics

All animal procedures were approved by the Institutional Animal Care and Use Committee. Male C57BL/6 mice (seven weeks old, weighing 20–25 g) were purchased from Hunan Silaike Jingda Experimental Animal Co., Ltd. (Changsha, China) and housed in a specific pathogen-free barrier facility at the Animal Experiment Center of Tongji Hospital, Tongji Medical College, Huazhong University of Science and Technology. The housing conditions maintained a 12-h light/dark cycle, a constant temperature of 22 °C, and provided free access to standard chow and sterilized water. Euthanasia was performed *via* inhalation of a high concentration of carbon dioxide. Death was confirmed by the absence of a pupillary reflex and cardiac arrest for over 3 min. Animal carcasses were sealed in dedicated yellow biohazard bags for unified disposal by the hospital’s animal center.

After one week of acclimatization, mice were randomly divided into a control group and an experimental group (*n* ≥ 3) based on body weight using a random number table. The stone model group received a daily intraperitoneal injection of glyoxylate (100 mg/kg) for one week, while the control group received an equivalent volume of normal saline. One week later, mice were euthanized, and kidneys were harvested. One kidney from each mouse was fixed, paraffin-embedded, sectioned, and subjected to antigen retrieval. The sections were then incubated overnight at 4 °C with a primary antibody against transferrin receptor 1 (TfR1, ABclonal, Cat# A5865) at a dilution of 1:100. This was followed by incubation with a corresponding secondary antibody (SeraCare, Cat# 5220-0336) at a dilution of 1:500 for 50 min at room temperature. Immunohistochemistry results were semi-quantitatively analyzed using ImageJ software. The analysis was performed independently by two investigators to ensure consistency.

Western blotting was employed to detect the expression of key ferroptosis-related proteins in renal tissue. Kidney tissues were homogenized in RIPA lysis buffer (Boster Biological Technology, Cat# AR0102) containing protease and phosphatase inhibitors. After low-temperature centrifugation, the supernatant was collected, boiled for denaturation, and separated *via* 10% SDS-PAGE gel electrophoresis. Proteins were subsequently transferred onto PVDF membranes using a wet transfer method. The membranes were blocked with Rapid Blocking Buffer (Boster Biological Technology, Cat# AR0041) for 1 h at room temperature, followed by overnight incubation at 4 °C with the following primary antibodies: anti-GPX4 (Huabio, Cat# ET1706-45), anti-SLC7A11 (Huabio, Cat# HA721868), and anti-β-Actin (Huabio, Cat# HA722023) as the loading control. Subsequently, the membranes were incubated with an HRP-conjugated secondary antibody (Servicebio, Cat# GB23303) for 1 h at room temperature. Protein bands were visualized using an ECL chemiluminescence kit (Boster Biological Technology, Cat# AR1191) and semi-quantitatively analyzed by measuring band density with ImageJ software. The relative expression level of each target protein was expressed as the grayscale ratio to β-Actin.

The ferrous ion (Fe^2+^) content in renal tissue was measured using a commercial assay kit (Solarbio, Cat# BC5415). The level of malondialdehyde (MDA), a marker of lipid peroxidation, was determined using an MDA assay kit (Beyotime, Cat# S0131S).

### Statistical methods

Continuous variables are presented as weighted median with interquartile range. Categorical variables are presented as unweighted counts with weighted percentages. Differences in continuous and categorical variables between groups were assessed using weighted linear regression and weighted Chi-square tests, respectively. Multivariable logistic regression analysis was employed to evaluate the associations between various iron load indicators and the risk of kidney stones. Three models were constructed: Model 1 was unadjusted; Model 2 was adjusted for age and sex; Model 3 was further adjusted for age, sex, BMI, race/ethnicity, and smoking status. All analyses of NHANES data accounted for the complex survey design and were performed using R software (version 4.3.2) with the corresponding survey-weighted R packages. ImageJ software was used for quantitative analysis. A two-sided *p* value of less than .05 was considered statistically significant.

## Results

### Basic characteristics

Among the 4,370 participants included in the analysis, 450 were kidney stone formers and 3,920 were non-formers, corresponding to an unweighted prevalence of 10.3%. After applying sample weights, the estimated prevalence among the U.S. adult population was 10.6%. Compared with non-formers, stone formers had a higher proportion of individuals who were older, had a higher BMI, and had a higher SIRI score. The risk of stone formation varied by race/ethnicity, with the highest prevalence observed in Non-Hispanic White individuals (14.7%) and the lowest in Non-Hispanic Black individuals (6.8%). Stone formers also exhibited significantly higher levels of sTfR and dietary iron intake, along with lower TSAT (*p* < .05 for all). No statistically significant differences were found between the two groups for other variables, including sex, smoking status, sedentary time, daily calcium intake, SF, SI, hemoglobin, and mean corpuscular hemoglobin (Table 1).

**Table 1.** Basic characteristics of stone and non-stone populations.

Characteristic	Overall *N* = 4,370^a^	No-stone *N* = 450^a^	Stone *N* = 3,920^a^	*p* Value^b^
Sex				.2
Female	2,259 (52%)	2,061 (53%)	198 (46%)	
Male	2,111 (48%)	1,859 (47%)	252 (54%)	
Age	48 (33, 62)	47 (32, 61)	57 (44, 68)	**<.001**
Age groups				**<.001**
20–39 Years	1,314 (36%)	1,236 (38%)	78 (19%)	
40–59 Years	1,386 (35%)	1,229 (34%)	157 (38%)	
60+ Years	1,670 (29%)	1,455 (28%)	215 (43%)	
Race				**.009**
Mexican American	588 (9.2%)	542 (9.5%)	46 (6.3%)	
Other Hispanic	409 (6.9%)	367 (7.0%)	42 (5.5%)	
Non-Hispanic White	1,596 (63%)	1,361 (62%)	235 (72%)	
Non-Hispanic Black	999 (11%)	931 (11%)	68 (7.1%)	
Other/multiracial	778 (10%)	719 (10%)	59 (9.2%)	
BMI	29 (25, 34)	29 (25, 34)	30 (26, 35)	**.005**
BMI groups				**.005**
Normal (<25)	1,108 (26%)	1,032 (27%)	76 (15%)	
Overweight(> =25,<30)	1,879 (44%)	1,647 (43%)	232 (51%)	
Obese(> =30)	1,383 (31%)	1,241 (30%)	142 (35%)	
Smoking				.5
Yes	1,871 (42%)	1,649 (42%)	222 (44%)	
No	2,499 (58%)	2,271 (58%)	228 (56%)	
Sedentary				.5
Sedentary time > 8h/day	1,226 (30%)	1,090 (30%)	136 (31%)	
Sedentary time < 8h/day	3,144 (70%)	2,830 (70%)	314 (69%)	
SIRI	1.08 (0.74, 1.58)	1.07 (0.73, 1.56)	1.11 (0.79, 1.70)	**.001**
Ferritin	104 (51, 190)	104 (52, 191)	105 (48, 180)	.5
Iron	83 (63, 106)	84 (63, 107)	78 (57, 102)	.056
Transferrin saturation	26 (20, 34)	26 (20, 34)	24 (17, 31)	**.019**
Transferrin receptor	2.87 (2.40, 3.52)	2.85 (2.38, 3.48)	3.05 (2.61, 3.74)	**.004**
Hemoglobin	14.20 (13.30, 15.10)	14.20 (13.30, 15.10)	14.30 (13.40, 15.20)	.2
Mean cell hemoglobin	33.70 (33.20, 34.20)	33.70 (33.20, 34.20)	33.70 (33.00, 34.20)	.4
Calcium intake	952 (648, 1,355)	947 (644, 1,348)	1,001 (689, 1,429)	.3
Iron intake	14 (10, 20)	13 (10, 20)	15 (10, 22)	**.030**

^a^ *N* (unweighted) (%); median (Q1, Q3).

^b^ Pearson’s X^2: Rao & Scott adjustment; design-based Kruskal–Wallis test.

Boldface indicates statistical significance (*p* < .05).

BMI: body mass index; SIRI: systemic inflammation response index.

### Iron status correlates with the risk of kidney stone development

In the unadjusted model (Model 1), the occurrence of kidney stones showed significant associations with SI (odds ratio [OR] = 0.94, 95% confidence interval [CI]: 0.89–0.99, *p* = .03), TSAT (OR = 0.79, 95% CI: 0.67–0.94, *p* = .011), sTfR (OR = 2.13, 95% CI: 1.24–3.67, *p* = .009), and daily iron intake (OR = 1.13, 95% CI: 1.06–1.20, *p* = .001). After adjusting for sex, age, race/ethnicity, and BMI (Model 2), these associations remained statistically significant (SI: OR = 0.92, 95% CI: 0.86–0.98, *p* = .035; TSAT: OR = 0.72, 95% CI: 0.59–0.89, *p* = .001; sTfR: OR = 2.64, 95% CI: 1.34–5.21, *p* = .017; iron intake: OR = 1.12, 95% CI: 1.04–1.20, *p* = .015). In the fully adjusted model (Model 3), which included sex, age, race/ethnicity, BMI, smoking status, sedentary time, daily calcium intake, and SIRI, TSAT and daily iron intake remained independently associated with kidney stone risk. Specifically, every 10% increase in TSAT was associated with a 26% lower likelihood of having kidney stones (OR = 0.74, 95% CI: 0.56–0.98, *p* = .04). Conversely, every 10 mg/day increase in dietary iron intake was associated with a 12% higher likelihood of kidney stones (OR = 1.12, 95% CI: 1.01–1.25, *p* = .036) (Table 2).

**Table 2.** Multi-model validation of the existence of a correlation between iron status and the risk of developing kidney stones.

Variables	Models	OR (95% CI)^a^	*p* Value
Ferritin	Model 1	1.0 (0.99, 1.00)	.2
	Model 2	0.99 (0.97, 1.00)	**.043**
	Model 3	0.98 (0.96, 1.00)	.091
Iron	Model 1	0.94 (0.89, 0.99)	**.030**
	Model 2	0.92 (0.86, 0.98)	**.035**
	Model 3	0.93 (0.85, 1.01)	.081
Transferrin saturation	Model 1	0.79 (0.67, 0.94)	**.011**
	Model 2	0.72 (0.59, 0.89)	**.010**
	Model 3	0.74 (0.57, 0.96)	**.040**
Transferrin receptor	Model 1	2.13 (1.24, 3.67)	**.009**
	Model 2	2.64 (1.34, 5.21)	**.017**
	Model 3	2.36 (0.95, 5.88)	.053
Hemoglobin	Model 1	1.52 (0.60, 3.85)	.4
	Model 2	0.79 (0.20, 3.09)	.8
	Model 3	0.76 (0.11, 5.22)	.9
Mean cell hemoglobin	Model 1	0.65 (0.11, 3.93)	.6
	Model 2	0.30 (0.03, 2.89)	.4
	Model 3	0.29 (0.02, 4.48)	.4
Iron intake	Model 1	1.13 (1.06, 1.20)	**.001**
	Model 2	1.12 (1.04, 1.20)	**.015**
	Model 3	1.12 (1.01, 1.24)	**.036**

*Notes:* Model 1: not adjusted for any covariates. Model 2: adjusted for age, sex, race, BMI. Model 3: included age, sex, race, BMI, smoking status, sedentary time, SIRI, calcium intake.

Boldface indicates statistical significance (*p* < .05).

^a^ OR = Odds Ratio, CI = Confidence Interval, for each ten-unit increase in the independent variable.

### Dose-response relationship between iron status and kidney stones

**Figure 2.**
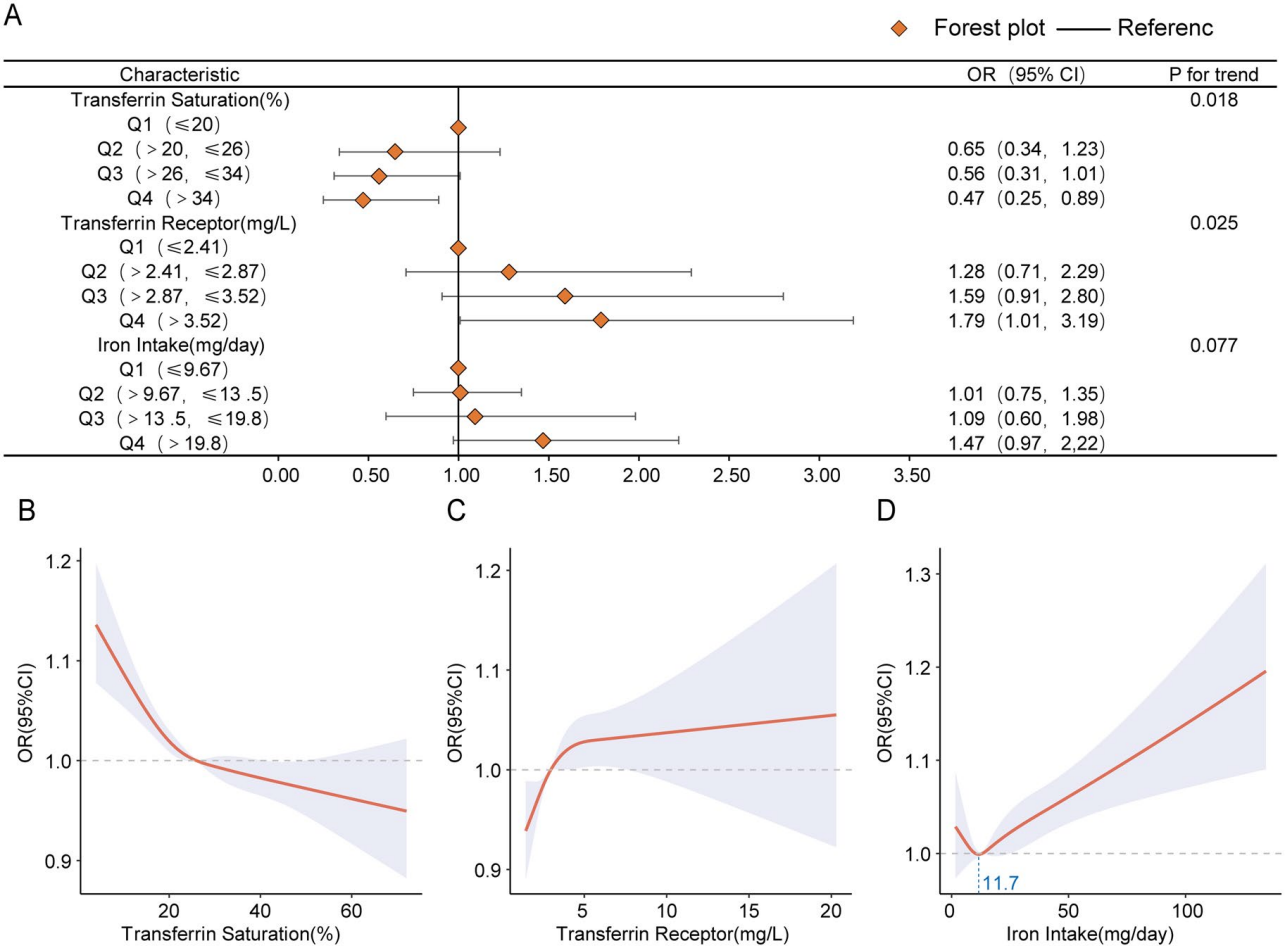
Dose–response relationships between iron status indicators and kidney stone risk. Forest plots illustrated the dose-response associations of TSAT, sTfR and dietary iron intake with kidney stone risk (A). RCS curves illustrate the nonlinear relationships of TSAT and sTfR with the risk of kidney stone formation (B,C). Moreover, RCS analysis revealed a U‑shaped (approximately hook‑shaped) association between dietary iron intake and kidney stone risk, with an inflection point at 11.7 mg/day (D).

To investigate the dose-response relationships between iron load indicators and kidney stones, the weighted study population was divided into quartiles based on each indicator, from lowest to highest. The lowest quartile served as the reference. A significant dose-response relationship was observed for TSAT (P for trend = 0.018) and sTfR (P for trend = 0.025). Although the risk of kidney stones tended to increase with higher daily iron intake, this trend did not reach statistical significance (P for trend = 0.077) (Figure 2(A)).

To further elucidate these relationships, restricted cubic spline (RCS) models were employed. Within the observed data range, TSAT showed a negative linear association with kidney stone risk (Figure 2(B)). In contrast, a positive association was observed for sTfR, with a potential inflection point at approximately 5.4 mg/L. Below this point, the risk increased markedly with rising receptor levels, above which the increase became less pronounced (Figure 2(C)).

Interestingly, the relationship between daily iron intake and kidney stone risk exhibited a non-linear, approximately U-shaped (or hook-like) pattern, with an inflection point at about 11.7 mg/day. Below this threshold, iron intake was inversely associated with kidney stone risk. Beyond this point, however, the risk increased significantly with higher iron intake (Figure 2(D)).

### Subgroup analyses

After applying sample weights and adjusting for all covariates, subgroup analyses were conducted based on sex, age, and BMI (Table 3). The results indicated that the associations between kidney stone risk and three iron load indicators – TSAT, sTfR, and daily iron intake – were not statistically significant among males. However, significant associations were observed among females (TSAT: OR = 0.67, 95% CI: 0.50–0.91, *p* = .022; sTfR: OR = 2.83, 95% CI: 1.12–7.19, *p* = .036; daily iron intake: OR = 1.15, 95% CI: 1.04–1.27, *p* = .020). Furthermore, these three indicators showed no significant association with kidney stone risk in subgroups aged 20–40 years or in those with a BMI < 25 kg/m^2^. In contrast, daily iron intake was positively associated with kidney stone risk among participants aged 40–60 years (OR = 1.20, 95% CI: 1.09–1.32, *p* = .006) and in those with a BMI ≥ 30 kg/m^2^ (OR = 1.19, 95% CI: 1.08–1.30, *p* = .007). However, tests for interaction did not reach statistical significance.

**Table 3.** Subgroup analysis of each iron status indicator in different population.

	Transferrin saturation (%)^a^	*p*-value	Transferrin receptor (mg/L)^a^	*p*-value	Iron intake (mg)^a^	*p*-value
**Sex**						
Male	0.80(0.61, 1.06)	0.093	0.096(0.09, 10.7)	0.9	1.08(0.82, 1.34)	0.6
Female	0.67(0.50, 0.91)	**0.022**	2.83(1.12, 7.19)	**0.036**	1.15(1.04, 1.27)	**0.02**
**Age**						
20–40	0.78(0.48, 1.26)	0.2	1.24(0.39, 3.98)	0.6	1.13(0.90, 1.41)	0.2
40–60	0.73(0.49, 1.09)	0.1	1.85(0.56, 6.11)	0.2	1.2(1.09, 1.32)	**0.006**
>60	0.75(0.54, 1.04)	0.07	6,26(0.6, 65.9)	0.1	1.06(0.89, 1.26)	0.4
**BMI**						
<25	0.94(0.62, 1.43)	0.7	2.92(0.09, 90.6)	0.4	1.07(0.83, 1.38)	0.5
25–30	0.71(0.50, 1.01)	0.054	3.08(0.86, 11)	0.071	1.06(0.86, 1.30)	0.5
>30	0.67(0.45, 1.02)	0.058	1.75(0.42, 7.38)	0.3	1.19(1.08, 1.30)	**0.007**

^a^OR(95% CI), *p*, for each ten-unit increase in the independent variable.

Boldface indicates statistical significance (*p* < .05).

### Animal study

**Figure 3.**
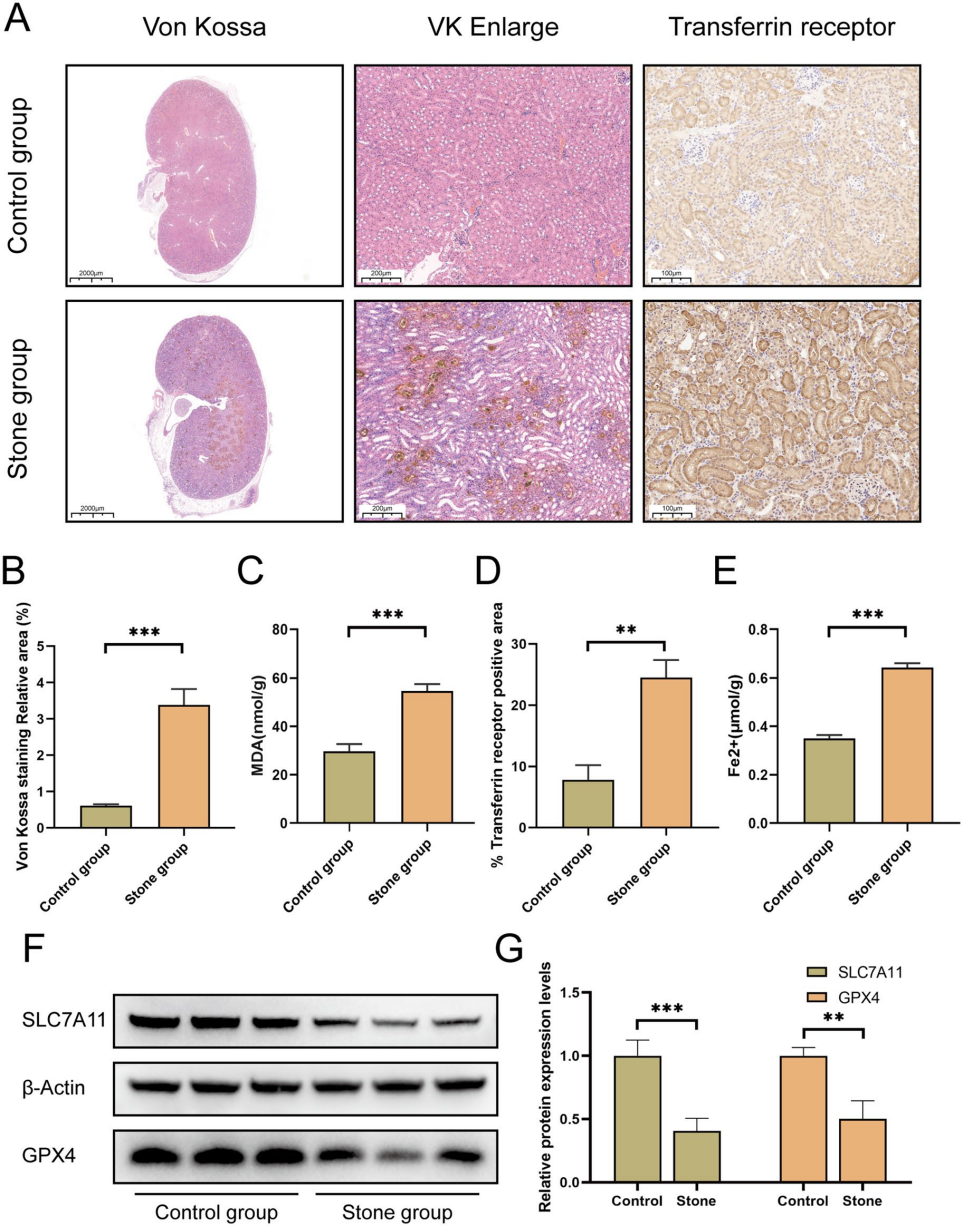
Increased iron deposition and ferroptosis in renal tissues of nephrolithiasis mice. Renal tissues from the stone group showed robust calcium salt deposition (Von Kossa staining) and elevated sTfR expression compared to the control group (A). Quantitative analyses confirmed greater Von Kossa-positive staining area, MDA levels, sTfR-positive area, and Fe²⁺ content in the stone group (B–E). Western blot analysis demonstrated enhanced ferroptosis activity in the stone group, as evidenced by downregulated SLC7A11 and GPX4 expression (F,G).

Immunohistochemical staining revealed that, compared with the control group, the kidney tissue of the stone model group exhibited a significantly larger area of calcium salt deposition, along with markedly enhanced expression intensity of sTfR in renal tubular epithelial cells (Figure 3(A)). Quantitative analysis based on Von Kossa staining and MDA immunofluorescence demonstrated that both the percentage area of calcium salt deposition and the fluorescence intensity of MDA were significantly higher in the stone model group (Figure 3(B,C); *p* < .001), indicating aggravated renal calcification and elevated lipid peroxidation. Semi-quantitative analysis of the sTfR-positive area using ImageJ software showed a significant expansion in the stone model group (Figure 3(D); *p* < .01), suggesting increased demand for renal iron transport. Furthermore, detection of Fe^2+^ content in renal tissue homogenates confirmed a significantly higher concentration in the stone model group (Figure 3(E); *p* < .001), providing direct evidence of iron accumulation. Subsequent Western blot analysis revealed downregulation of GPX4 and SLC7A11 expression in the stone model group (Figure 3(F)). Densitometric quantitative analysis further confirmed that these differences in protein expression were statistically significant (Figure 3(G); *p* < .001). Collectively, these findings suggest that local dysregulation of iron metabolism in renal tissue may be involved in the pathogenesis of kidney stones.

## Discussion

In this cross-sectional study involving 4,370 participants, we found significant associations between iron status indicators and the risk of kidney stones. These associations persisted after adjusting for covariates including sex, age, race, BMI, smoking status, sedentary behavior, daily calcium intake, and SIRI. Specifically, kidney stone risk was positively associated with sTfR and dietary iron intake, but negatively associated with TSAT. Furthermore, evidence from animal experiments indicated that, compared to the control group, the renal tissue of mice in the stone model group exhibited a more pronounced state of iron accumulation and a more active ferroptosis process. Our findings suggest that dysregulation of iron metabolism may play an important role in the formation and progression of kidney stones.

Iron is an essential trace element for maintaining normal cellular physiological functions. However, elevated iron load has been associated with various diseases, including cognitive dysfunction in the elderly and metabolic disorders such as liver fibrosis [17,18]. Excess iron can catalyze the Fenton reaction, leading to the accumulation of reactive oxygen species and increased oxidative stress, thereby causing cellular damage [19,20]. This pathophysiological cascade aligns closely with the known processes involved in kidney stone formation. Therefore, systemic or local abnormalities in iron status might be associated with stone formation, although current evidence in this area remains relatively limited. To our knowledge, this study is the first to systematically investigate the association between iron load indicators and kidney stone risk using a large-scale population database combined with an animal experimental model, aiming to provide a new scientific basis for the prevention and clinical management of nephrolithiasis.

Previous animal studies have reported elevated expression of iron transport proteins, such as ceruloplasmin and transferrin, in stone-forming model mice compared to controls, hinting at a potential role of high iron status in lithogenesis [21]. Interestingly, in that study, low TSAT and high sTfR, typical markers of ‘functional iron deficiency’ or ‘impaired iron utilization’, often indicate that circulatory iron supply cannot meet the demands of the bone marrow. This would seem to point to a conclusion that high iron load is negatively associated with kidney stone risk. However, the positive association we observed between iron intake and stone risk contradicts a simple ‘iron deficiency’ hypothesis. We hypothesize that, on one hand, kidney stones as a chronic inflammatory condition may directly induce functional iron deficiency (manifested as low TSAT and high sTfR) while simultaneously promoting the recruitment and deposition of iron in the kidney [22–24]. On the other hand, the ‘gut-kidney axis’ hypothesis may also explain this apparent paradox. Numerous studies have shown that kidney stone patients commonly exhibit abnormally increased intestinal oxalate absorption [25,26]. Iron that is ‘consumed’ to bind oxalate or abnormally diverted may fail to effectively enter the circulatory iron pool, leading to relative SI insufficiency. Concurrently, some iron may enter circulation in active forms such as non-transferrin bound iron and deposit in the kidney, resulting in local iron overload [27]. It must be emphasized, however, that while these explanations are pathophysiologically plausible, they remain speculative integrations based on cross-sectional data. Their validity and precise *in vivo* pathways require confirmation through further research. Future studies should aim to confirm the relationship between this pattern of iron metabolism and stone risk using more prospective cohorts and directly validate the existence of renal iron deposition and its functional role in stone formation through animal models or interventional studies. Notably, our study did not find significant associations between kidney stone risk and other indicators such as SF or hemoglobin. This may reflect the different pathophysiological significance of various iron metabolism markers. Ferritin, the major iron storage protein, is strongly influenced by inflammatory status. In a chronic inflammatory disease like nephrolithiasis, inflammation associated with stones may elevate baseline ferritin levels [28]. Hemoglobin primarily reflects the efficiency of bone marrow iron utilization for erythropoiesis, a more downstream indicator that may be insensitive to changes in local renal iron metabolism [29]. In contrast, TSAT and sTfR appear to more sensitively reflect circulatory iron availability and tissue iron demand, which is where the main associations in our study were concentrated [30]. Therefore, these findings collectively suggest that, compared to systemic iron storage or hematopoietic utilization, circulatory iron transport and its dysregulation may be more directly associated with kidney stone risk.

Consistent with the above speculation, animal experiments provided direct evidence for the ‘local renal iron overload’ hypothesis. We found that in the renal tissue of stone-forming mice, the expression of transferrin receptor was significantly upregulated, and Fe^2+^ content was also markedly increased. This suggests that under stone-forming conditions, the kidney may undergo a microscopic process of ‘active iron recruitment’, leading to the formation of a ‘local toxic iron pool’. This finding provides an experimental basis for explaining the macroscopic paradox of coexisting systemic iron utilization impairment and local renal iron overload. Notably, sTfR is also a key biomarker of ferroptosis, and its elevated expression implies enhanced cellular iron status and increased ferroptosis activity [31–33]. Western blot analysis further confirmed that the expression levels of anti-ferroptosis proteins GPX4 and SLC7A11 were downregulated in the kidney stone model group. These findings indicate that local iron overload may activate the ferroptosis pathway. Ferroptosis, as an iron-dependent novel form of cell death, may theoretically exacerbate damage to renal tubular epithelial cells, thereby creating conditions for stone formation [34,35]. These results may provide a new entry point for investigating the specific mechanisms by which iron metabolism imbalance contributes to kidney stone formation.

Dietary iron is the primary source of iron in the human body. Multiple studies have indicated that both excessive and insufficient iron intake are associated with various diseases [36–38]. In this study, kidney stone risk was positively associated with iron intake. Increased iron intake may elevate systemic oxidative stress levels and disrupt iron homeostasis, thereby contributing to the onset of various diseases [39]. Interestingly, the RCS curve revealed that the relationship between iron intake and stone risk was not a simple linear one. Within a lower range, stone risk decreased with increasing iron intake, reaching its lowest point at 11.7 mg/day. As an essential metal involved in numerous critical cellular metabolic processes, iron may be deficient in various disease states [40,41]. It is noteworthy, however, that 50% of the US population has a daily intake exceeding 13.5 mg, suggesting that a substantial portion of the population may be in a state of relative iron overload.

Subgroup analyses suggested that the associations between iron metabolism indicators and stone risk were concentrated primarily among females, while the risk associated with dietary iron intake was more pronounced in individuals aged 40–60 and those with obesity. However, formal tests for interaction did not reach statistical significance. Future studies with larger sample sizes are needed to further validate these potential differential patterns.

To our knowledge, this is the first cross-sectional study to investigate the relationship between iron load and kidney stone risk. By utilizing NHANES data and conducting animal experiments, we have identified an association between human iron load and kidney stone formation, offering a new perspective for prevention and management. However, this study has several limitations. First, the cross-sectional design cannot establish a causal relationship between iron status and kidney stone risk. Moreover, NHANES does not record the specific timing of stone events, making it difficult to clarify the temporal sequence between serum indicators and stone formation. Second, the study population is limited to U.S. adults, and the generalizability of the conclusions needs to be verified in other populations. Third, methodological aspects warrant consideration: 1) Dietary iron analysis was not energy-adjusted, primarily due to the high collinearity between total energy intake and the key covariate BMI already included in the model. Additionally, constrained by data structure, we were unable to distinguish the sources of iron. Future studies require more detailed dietary data to explore this issue. 2) Other important dietary confounding factors could not be adjusted for due to data unavailability, and residual confounding may affect effect estimates. 3) The animal experiment had a relatively small sample size. The robustness of the conclusions awaits validation through larger-scale, rigorously designed experiments. Finally, key iron indicators were only intensively measured in the NHANES 2017–2018 cycle, limiting our ability to perform validation analyses in larger populations or across different time periods. In conclusion, this study proposes a novel direction in the pathogenesis of kidney stones: patients may exhibit a paradoxical metabolic state characterized by coexisting systemic relative iron deficiency and local renal iron overload. This finding offers a new perspective for understanding the pathology of stone formation. Future prospective cohort studies are needed to validate the causal relationship between this metabolic pattern and stone risk, and interventional research should be conducted to elucidate the underlying mechanisms and clinical implications.

## Summary

This study presents the first integrated analysis of epidemiological data from the NHANES and *in vivo* animal models to systematically investigate the association between iron metabolism dysregulation and kidney stone risk. Population-based analyses revealed that increased dietary iron intake and decreased TSAT were independently associated with a higher risk of kidney stones. Animal experiments further demonstrated that stone formation was accompanied by local renal iron accumulation, elevated oxidative stress, and activation of key ferroptosis pathways. Collectively, these findings suggest that both systemic and local renal iron metabolism abnormalities may play a significant role in the pathogenesis of kidney stones, providing a novel perspective for understanding the disease and laying preliminary scientific groundwork for future preventive strategies targeting iron metabolism or ferroptosis pathways.

## Supplementary Material

Supplemental Table 1.docx

## Data Availability

The National Health and Nutrition Examination Survey (NHANES) data used in this study are publicly available from the Centers for Disease Control and Prevention (CDC) website (https://www.cdc.gov/nchs/nhanes/). The original data generated from the animal experiments and the analyzed datasets during this study are available from the corresponding author on reasonable request.
