# Supplementary material for: Dysregulated iron metabolism and kidney stone risk: an epidemiological and experimental study
Source: Ren Fail. 2026 Mar 3;48(1):2631316. doi: 10.1080/0886022X.2026.2631316 (PMC12958378; doi:10.1080/0886022X.2026.2631316)
Supplement: Supplemental Table 1.docx [file IRNF_A_2631316_SM0763.docx]

**Supplemental Table 1：Characteristics of NHANES participants, 2017-2018**

| **Characteristic** | **Overall**  N = 461,970,859^1^ | **Weighted U.S. adult population**  N = 223,233,320^1^ | **Population in this study**  N =23,684,178^1^ |
| --- | --- | --- | --- |
| Stone.group |  |  |  |
| *No-stone* | 86521 (89%) | 3920 (89%) | 4732 (89%) |
| *Stone* | 983 (11%) | 450 (11%) | 533 (11%) |
| **Sex** |  |  |  |
| *Female* | 4983 (52%) | 2259 (52%) | 2724 (52%) |
| *Male* | 4652 (48%) | 2111 (48%) | 2541 (48%) |
| **Age** | 48 (33, 62) | 48 (33, 62) | 48 (33, 62) |
| **Age.group** |  |  |  |
| *20-39 years* | 2903 (36%) | 1314 (36%) | 1589 (36%) |
| *40-59 years* | 3044 (35%) | 1386 (35%) | 1658 (35%) |
| *60+ years* | 3688 (29%) | 1670 (29%) | 2018 (29%) |
| **Race** |  |  |  |
| *Mexican American* | 1286 (9.0%) | 588 (9.2%) | 698 (8.9%) |
| *Other Hispanic* | 905 (6.9%) | 409 (6.9%) | 496 (6.9%) |
| *Non-Hispanic White* | 3403 (62%) | 1596 (63%) | 1807 (62%) |
| *Non-Hispanic Black* | 2239 (11%) | 999 (11%) | 1240 (12%) |
| *Other/multiracial* | 1802 (10%) | 778 (10%) | 1024 (11%) |
| **BMI** | 29 (25, 34) | 29 (25, 34) | 29 (25, 34) |
| **BMI.group** |  |  |  |
| *Normal( <25)* | 2445 (26%) | 1108 (26%) | 1337 (26%) |
| *Overweight(>=25,<30)* | 4050 (44%) | 1879 (44%) | 2171 (43%) |
| *Obese(>=30)* | 3050 (31%) | 1383 (31%) | 1667 (31%) |
| **Smoke** |  |  |  |
| *Yes* | 4080 (42%) | 1871 (42%) | 2209 (42%) |
| *No* | 5555 (58%) | 2499 (58%) | 3056 (58%) |
| **Sedentary** |  |  |  |
| *Sedentary Time>8h/day* | 2700 (30%) | 1226 (30%) | 1474 (30%) |
| *Sedentary Time<8h/day* | 6925 (70%) | 3144 (70%) | 3781 (70%) |
| **SIRI** | 1.08 (0.74, 1.58) | 1.08 (0.74, 1.58) | 1.08 (0.74, 1.58) |
| **Ferritin** | 104 (52, 190) | 104 (51, 190) | 104 (52, 190) |
| **Iron** | 83 (63, 106) | 83 (63, 106) | 83 (63, 106) |
| **Transferrin saturation** | 26 (20, 34) | 26 (20, 34) | 26 (20, 34) |
| **Transferrin receptor** | 2.87  (2.40, 3.52) | 2.87  (2.40, 3.52) | 2.87  (2.40, 3.53) |
| **Hemoglobin** | 14.20  (13.30, 15.10) | 14.20  (13.30, 15.10) | 14.20  (13.30, 15.10) |
| **Mean cell hemoglobin** | 33.70  (33.20, 34.20) | 33.70  (33.20, 34.20) | 33.70  (33.20, 34.20) |
| **Calcium intake** | 951  (647, 1,349) | 952  (648, 1,355) | 949  (646, 1,346) |
| **Iron intake** | 14 (10, 20) | 14 (10, 20) | 14 (10, 20) |
| ^1^N (weighted) (%); Median (Q1, Q3) | | | |
